# Family of microRNA-146 Regulates RARβ in Papillary Thyroid Carcinoma

**DOI:** 10.1371/journal.pone.0151968

**Published:** 2016-03-24

**Authors:** Agnieszka Anna Czajka, Anna Wójcicka, Anna Kubiak, Marta Kotlarek, Elwira Bakuła-Zalewska, Łukasz Koperski, Wiesław Wiechno, Krystian Jażdżewski

**Affiliations:** 1 Genomic Medicine; Medical University of Warsaw, Warsaw, Poland; 2 Laboratory of Human Cancer Genetics, Centre of New Technologies, CENT, University of Warsaw, Warsaw, Poland; 3 Department of Pathology, Maria Skłodowska-Curie Memorial Cancer Center–Institute of Oncology, Warsaw, Poland; 4 Department of Pathology, Medical University of Warsaw, Warsaw, Poland; 5 Department of General and Thoracic Surgery, Medical University of Warsaw, Warsaw, Poland; University of Texas, MD Anderson Cancer Center, UNITED STATES

## Abstract

Retinoic acid is a promising tool in adjuvant cancer therapies, including refractory thyroid cancer, and its biological role is mediated by the retinoic acid receptor beta (RARβ). However, expression of RARβ is lowered in papillary thyroid carcinoma (PTC), contributing to promotion of tumor growth and inefficiency of retinoic acid and radioactive iodine treatment. The causes of aberrant *RARB* expression are largely unknown. We hypothesized that the culpable mechanisms include the action of microRNAs from the miR-146 family, previously identified as significantly upregulated in PTC tumors. To test this hypothesis, we assessed the expression of *RARB* as well as miR-146a-5p and miR-146b-5p in 48 PTC tumor/normal tissue pairs by Taqman assay to reveal that the expression of *RARB* was 3.28-fold decreased, and miR-146b-5p was 28.9-fold increased in PTC tumors. Direct interaction between miRs and *RARB* was determined in the luciferase assay and further confirmed in cell lines, where overexpression of miR-146a-5p and miR-146b-5p caused a 31% and 33% decrease in endogenous *RARB* mRNA levels. Inhibition of miR-146a and miR-146b resulted in 62.5% and 45.4% increase of *RARB*, respectively, and a concomitant decrease in proliferation rates of thyroid cancer cell lines, analyzed in xCELLigence system.We showed that two microRNAs of the miR-146 family directly regulate *RARB*. Inhibition of miRs resulted in restoration of *RARB* expression and decreased rates of proliferation of thyroid cancer cells. By restoring *RARB* levels, microRNA inhibitors may become part of an adjuvant therapy in thyroid cancer patients.

## Introduction

Papillary thyroid carcinoma (PTC) is the most common endocrine malignancy [1]. About half of PTC cases are associated with presence of the RET/PTC rearrangement resulting in aberrances of RET/PTC-RAS/BRAF pathway. The most common mutation within the pathway is the BRAF V600E, occurring in approximately 44–60% of PTC tumors [2,3]. The BRAF mutation is associated with a lower sensitivity to radioiodine therapy, due to reduced efficiency of iodine uptake [4,5]. However, many PTC cases do not exhibit any known somatic mutation and the molecular mechanisms responsible for the initiation and progression of cancer in the thyroid gland remain unexplained [6].

A significant number of human cancers exhibit aberrant expression of *RARB* gene [7–9]. Retinoic acid, a ligand for RARβ is an analogue of vitamin A (retinol) that plays important role in regulation of growth, differentiation and apoptosis. Due to its regulatory function retinoic acid has been proposed a promising target for adjuvant cancer therapies, including the therapy of refractory thyroid cancer [10]. RARβ regulates essential pathways associated with the tumor-suppressive effects of retinoids in various cells [11]. It is believed that changes in RARβ expression underlie neoplastic transformation, and inactivation of RARβ was demonstrated in lung, head and neck, as well as in mammary tumors [7–9]. In most thyroid carcinomas the retinoic acid receptors are downregulated, and the responsible mechanisms have not yet been fully elucidated. [12].

MicroRNAs (miRNAs, miRs) are short, non-coding RNAs that function as negative regulators of expression of protein-encoding genes by annealing to complementary sequences in 3’ untranslated regions (3’UTRs) of mRNAs and inhibiting further steps of protein synthesis [13]. In our previous studies we found severe deregulation of microRNAs in papillary thyroid carcinoma with miR-146a-5p and miR-146b-5p being on top of the list of up-regulated genes [14,15]. Moreover, the genetic variant of miR-146a has been proved to increase the risk of acquiring PTC [16,17]. While studying the function of the miR-146 family, we noticed that both, miR-146a-5p and miR-146b-5p, putatively regulate the expression of *RARB*. We hypothesized that the loss of the expression of *RARB* in thyroid cancer tissue might be a result of the up-regulation of the miR-146a family, and the inhibition of these miRs might lead to the restoration of RARβ and increased effectiveness of retinoic acid adjuvant therapy.

## Materials and Methods

### Thyroid Tissue Samples

Samples from PTC tumors (PTC-T, n = 48) and paired unaffected thyroid tissue (PTC-N, n = 48) were obtained from patients who underwent surgical resection at the Medical University of Warsaw, Poland. The study was approved by the Institutional Review Board at the Medical University of Warsaw and written consent was obtained from each of the enrolled patients. Fresh tissue samples were snap-frozen on dry ice and stored at −80°C.

### Nucleic Acid Extraction and Quantification

Total RNA was extracted from tissue samples using TRIZOL solution (Invitrogen, Life Technologies, Carlsbad, USA). Total RNA from cell lines was extracted using GeneMATRIX Universal RNA/miRNA Purification Kit (EURx, Gdansk, Poland). Integrity of RNA was assessed by NanoDrop2000 (Thermo Scientific, Wilmington, Delaware USA). RNA was reverse transcribed into cDNA with the Reverse Transcriptase M-MLV (Promega, Madison, Wisconsin, USA). Real–Time PCR was performed using TaqMan Universal Master Mix II (Life Technologies, Carlsbad, USA) on the LightCycler 480 (Roche, Mannheim, Germany) under the following conditions: an initial 10 min denaturation period at 95°C followed by 45 cycles of denaturation in 95°C for 10 sec, annealing at 60°C for 30 sec and extension at 72°C for 1 sec. The TaqMan probes for *RARB*, miR-146a-5p, miR-146b-5p, miR-421-5p, U6B and *GAPDH* were purchased from Life Technologies (Carlsbad, USA). U6B and *GAPDH* were employed for normalization, and the amount of each transcript was quantified based on the 2^−ΔCT^ formula. Ct values for both genes are given in S1 Table.

### Cell Culture and Reagents

Non-small cell lung carcinoma (A549, ATCC) and human cervical cancer (HeLa, ATTC) cell lines were cultured in DMEM supplemented with 10% FBS and 1% Penicillin/Streptomycin (100ug/ml) at 37°C in a humified atmosphere of 5% CO_2._ A549 cells used for microRNA inhibitors study were treated with 1μM all-trans retinoic acid (ATRA) for 7 days to induce the expression of *RARB* gene (S1 Fig). K1 cell line (human papillary thyroid carcinoma) [18] was cultured in DMEM + HAM’sF12 + MDCB105 (in 2:1:1 ratio) with 10% FBS, 4mM L-Glutamine and Penicillin/Streptomycin (100ug/ml) at 37°C in a humified atmosphere of 5% CO_2._ HeLa cells were used for the luciferase reporter assay. For all the cell lines, *RARB* and microRNA expression levels were quantified (S1 Fig).

### Plasmid Constructs

The 3’UTR of the human *RARB* gene was amplified from human genomic DNA using specific primers (S2 Table) and cloned into the Xba1 site of pGL3-Control vector to yield pGL3-RARB plasmid. To obtain microRNA expression plasmids, genomic DNA encoding for miR-146b and miR-421 (control, with no binding sites in *RARB* 3’UTR) was amplified and inserted into the pcDNA3 plasmid to yield pcDNA3-miR-146b or pcDNA3-miR-421 (control vector), respectively. pcDNA3-miR-146a plasmid was obtained previously [17]. To obtain a plasmid expressing only functional miR-146a-5p we mutated the seed region of miR-146a-3p strand using targeted mutagenesis (S2 Table). pGL3-sponge-miR was prepared following the method described by [19]. An 80-nt long oligonucleotide containing two tandem sequences complementary to the microRNA was synthesized for miR-146a-5p and miR-146b-5p. Each template was amplified with specific primers containing restriction sites for PstI and KpnI and cloned into the pGL3 control vector to yield pGL3-sponge-miR-146a and pGL3-sponge-miR-146b plasmids (S2 Table).

### Luciferase Reporter Assays

HeLa cells with low endogenous expression of *RARB* and both miRs of miR-146 family were plated at 7.5x10^4^ cells per well in a 12-well plate, and 24h later cotransfected with 800ng of pGL3-RARB vector, 60ng of pRL-TK vector (transfection control) and 400ng of an individual microRNA vector (pcDNA3-miR-146a-5p, pcDNA3-miR-146b, pcDNA3-miR-421) using FuGene HD reagent (Roche, Mannheim, Germany). After 24h cells were washed with PBS and lysed, then the Dual-Luciferase Reporter 1000 Assay system was used to measure the luciferase activity on a GloMax Multi Detection System (Promega, Madison, Wisconsin, USA). All data are represented as relative fold change of firefly luciferase activity normalized to Renilla luciferase activity. All experiments were performed in triplicates.

### Inhibition of Endogenous *RARB* Expression

For mRNA analysis, A549 cells were seeded onto 12-well plate at 7.5x10^4^ and after 24h transfected with 500ng of each plasmids (pcDNA3-miR-146a-5p, pcDNA3-miR-146b, pcDNA3-miR-421) using FuGene HD reagent (Roche, Mannheim, Germany). Cells were cultured for 36h, washed with PBS and lysed with the GeneMATRIX Universal RNA/miRNA Purification Kit (EURx, Gdansk, Poland) lysis buffer. For protein analysis, A549 cells were seeded onto 6-well plate at 1.5x10^5^ and after 24h transfected with 1000ng of each plasmids (pcDNA3-miR-146a-5p, pcDNA3-miR-146b, pcDNA3-miR-421) using FuGene HD reagent (Roche, Mannheim, Germany). Cells were cultured for 72h, washed with PBS and lysed with homogenization buffer. All experiments were performed in triplicates.

### Protein Extraction and Western Blot Analysis

After lysis in homogenization buffer (20mM Tris-HCl pH = 7,4; 5mM EGTA; 2mM EDTA; 150mM NaCl; 1% TritonX-100; 10μM Leupeptin; 0.5mM PMSF) at 4°C cells were centrifuged, and protein concentration in supernatant was measured with Bradford reagent (Sigma, Saint Louis, Missouri, USA) in GloMax Multi Detection System (Promega, Madison, Wisconsin, USA). 50μg of total protein extracts were electrophoresed in a 6–10% gradient SDS–acrylamide gels in room temperature and transferred to PVDF membranes. The membranes were blocked overnight at 4°C in 5% non–fat dry milk in Tris buffered saline containing 0,1% Tween20 (TBST, pH = 7.6). For immunodetection, membranes were incubated with specific antibodies in TBST buffer: primary antibody for RARβ –rabbit monoclonal retinoic acid receptor beta antibody (clone EPR2017; NBP1-96734 Novus Biologicals, Littleton, Colorado, USA) (2h incubation), for β-actin–rabbit polyclonal antibody (GTX110564; GeneTex, Irvine, California, USA) (2h incubation), and subsequently with secondary antibody: horseradish peroxidase-conjugated goat polyclonal anti-rabbit IgG (bs-0295G-HRP; Bioss, Woburn, Massachusetts) (1.5h incubation). After immunodetection, membranes were washed 3x in TBST buffer and exposed to a chemiluminescence reagent (Coumaric acid, H_2_O_2_, luminol in 1M TrisHCl, pH = 8.5). The amount of specific protein was estimated densitometrically after normalization to expression of β-actin.

### Cell Proliferation Analysis

K1 cell lines were transfected with pGL3-sponge-miR-146a, pGL3-sponge-miR-146b, or an empty pGL3-Control vector and proliferation was analyzed using the xCELLigence system (ACEA Biosciences, San Diego, California, USA) measuring the electrical resistance of cells that adhere to electrodes on the surface. Resistance was measured every 30 minutes for 80 hours. The final Cell index values show the difference between the resistance generated by the cells in each time point and the resistance of the medium without cells.

### MicroRNA Inhibition

For the analysis of microRNA inhibition, K1 cells were seeded on a 12-well plate at 6.5x10^4^ and 24h later cotransfected with 1μg of pGL3-sponge-miR-146a, 1μg of pGL3-sponge-miR-146b, or an empty pGL3-Control vector (control of sponge activity) and 80ng of pRL-TK vector (transfection control) using FuGene HD reagent (Roche, Mannheim, Germany). After 24 hrs cells were analyzed in the luciferase assay. Total RNA from lysates was extracted with Trizol solution and served for the analysis of *RARB* expression in a Real-Time PCR reaction.

### *In Silico* and Statistical Analyses

MicroRNAs targeting *RARB* were identified in silico using TargetRank, TargetScan and miRecords. Statistical analysis of the obtained data was performed in GraphPad Prism. Data distribution was analyzed using the Shapiro-Wilk test. Normally distributed data were analyzed using t-test, and nonparametric data by Wilcoxon test. *P* <0.05 was considered statistically significant.

## Results

### Expression of *RARB* Is Reduced in Papillary Thyroid Carcinoma

**Fig 1:**
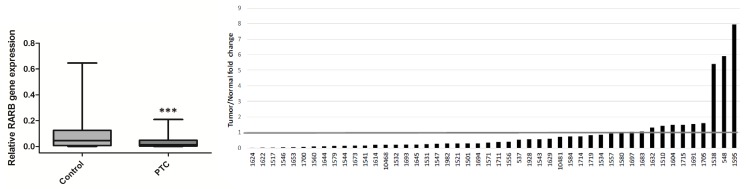
Expression of *RARB* mRNA levels in PTC tumors relative to paired control tissue (n = 48), normalized to the reference gene *GAPDH*. SQ-PCR reaction for each sample was performed in triplicate.

Levels of *RARB* mRNA were determined in 48 pairs of PTC and control samples using real-time PCR. *RARB* expression was reduced 3.28-fold in PTC tumors compared to control tissues (p<0.0001) (data not shown). Lowered levels of *RARB* mRNA were observed in 38 (77%) tumor samples (Fig 1).

### Expression of miR-146b Is Elevated in Papillary Thyroid Carcinoma

**Fig 2:**
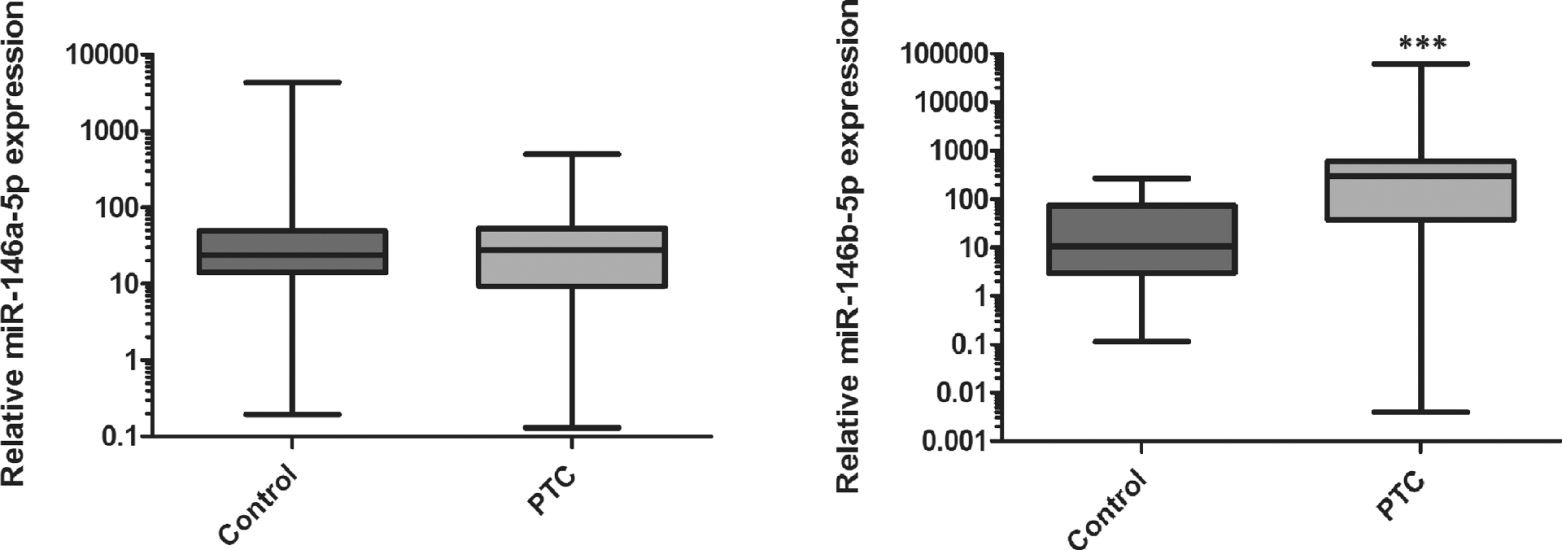
Expression of miR-146a-5p (A) and miR-146b-5p (B) in PTC tumors and matched control tissue from the same patient (n = 48), normalized by the reference gene *RNU6B*. The data are medians +/- SD (95% CI). Statistical analysis was performed using Wilcoxon paired test (***P <0,001).

Levels of expression of miR-146a and miR-146b were determined in 48 pairs of PTC and control samples using real-time PCR. The expression of miR-146b-5p was 28.9-fold higher in PTC tumors compared to control tissue (p < 0.0001, Fig 2). We found no differences in expression of miR-146a-5p between PTC tumors and control tissues (p = 0.78).

### Sequence of the 3’UTR of *RARB* Contains Putative Target Sites for miR-146a and miR-146b

**Fig 3:**
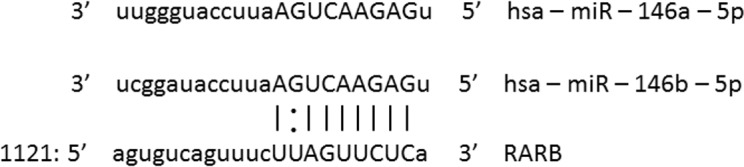
Positions of putative miRNA binding sites in *RARB* 3′UTR identified by TargetScan and RNAHybrid. miRNAs binding to 3′UTRs requires only partial complementarity to the target. Nucleotides of perfect complementarity are shown as seed match.

*In silico* analysis revealed that the 3’UTR of human *RARB* gene harbors putative miRNA binding sites for miR-146a and miR-146b (Fig 3). All binding sites are broadly conserved among vertebrates.

### miR-146a and miR-146b Directly Target *RARB* 3’UTR

**Fig 4:**
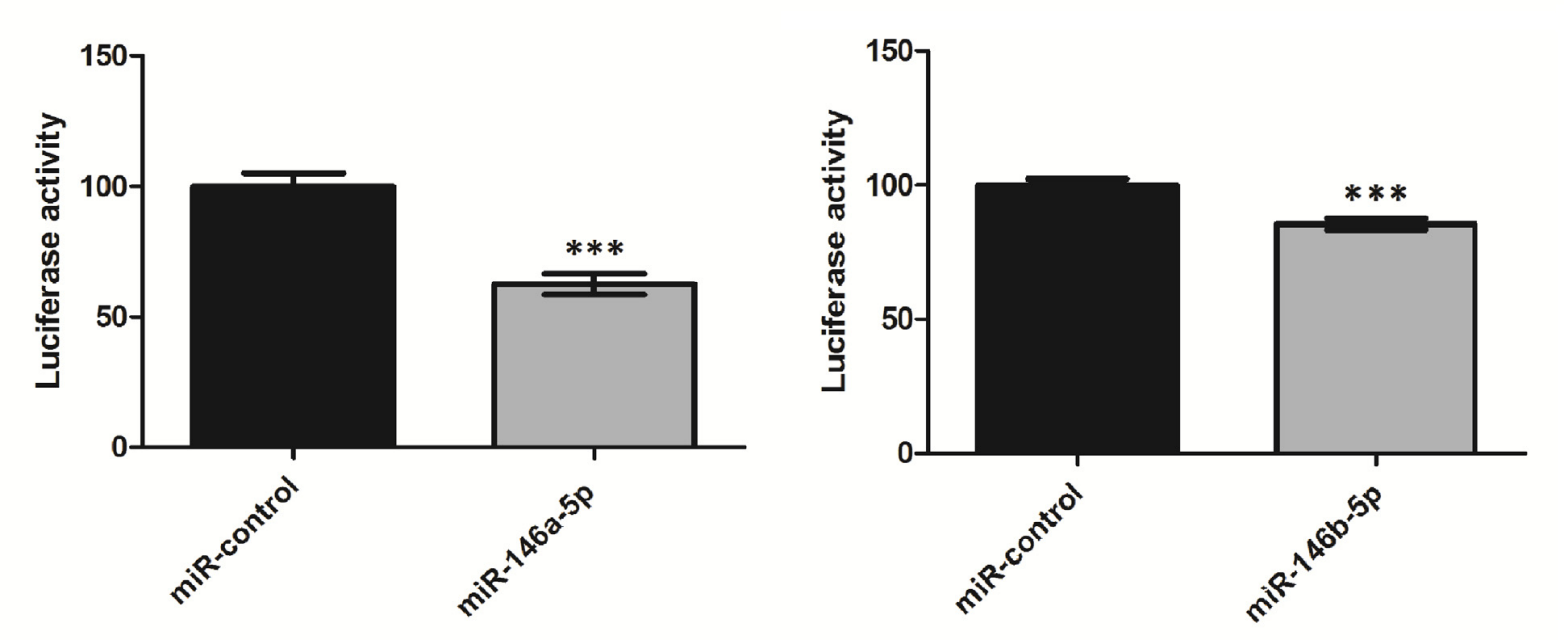
Luciferase activity in transfected HeLa cells. HeLa cells transfected with pGL3-3'UTR-RARB and pcDNA3-miR-146a-5p (A) or pcDNA3-miR-146b-5p (B). Luciferase activity is showed as a percentage relative to the control (cells transfected with pcDNA3-miR-421). The results are normalized by Renilla luciferase and derive from three experiments, each performed in triplicates. The graph shows the mean, along with deviations from mean (SEM). Statistical analysis was performed using an unpaired t test (*** *P*<0.001).

In order to analyze direct binding of miR-146a and miR-146b to the 3’UTR of *RARB* gene, HeLa cell line was cotransfected with a reporter pGL3-RARB plasmid and microRNA expression vector: pcDNA3-miR-146a-5p or pcDNA3-miR-146b-5p. The induction of microRNA expression was confirmed in a Real–Time PCR assay (S1 Fig). Transfection of cells with pcDNA3-miR-146a-5p and pcDNA3-miR-146b-5p resulted in a 37% (p = 0.00001) and 15% (p = 0.0001) reduction of luciferase activity, respectively, compared to control pcDNA3-miR-421 (miR-control) (Fig 4).

### miR-146a and miR-146b Inhibit Endogenous *RARB* Expression in Cancer Cell Lines

**Fig 5:**
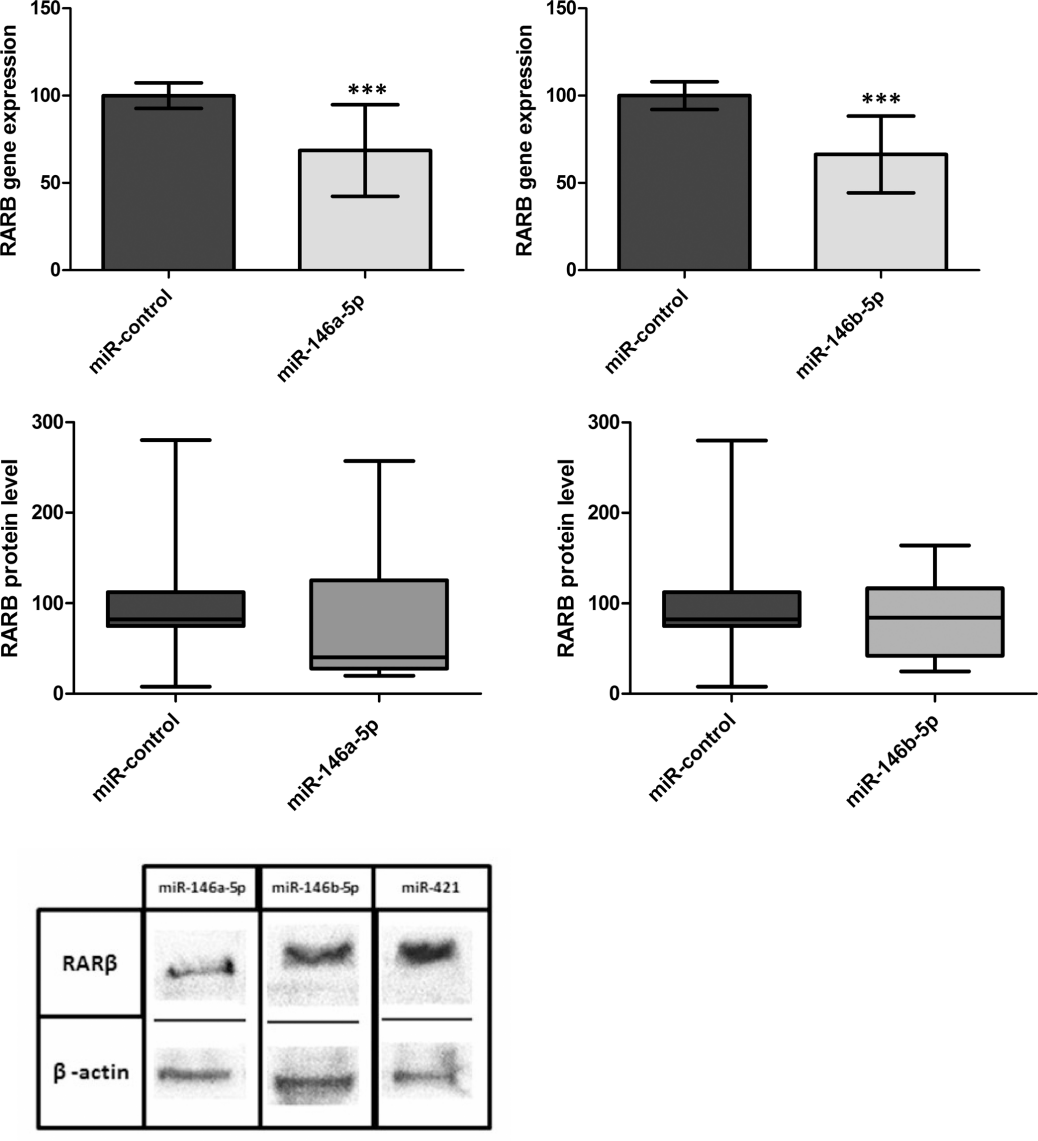
Reduction of endogenous RARB mRNA is shown by real-time PCR Taqman assay in the A549 cell line transfected with pcDNA3-miR-146a-5p (A) or pcDNA3-miR-146b-5p (B) or control plasmid. Data are expressed as mean values +/- SEM. Statistical analysis was performed using an unpaired t test (*** p <0.001). Western blotting for RARβ and β-actin (loading control) in the A549 cells transfected with pcDNA3-miR-146a-5p (C), pcDNA3-miR-146b-5p (D) or control plasmid has shown no significant reduction of protein level. Cells were harvested 72 h after transfection; the relative density of bands was quantified by densitometry.

To analyze influence of miR-146a and miR-146b on endogenous *RARB* expression, A549 cells with relatively high *RARB* levels were transfected with miR-146a, miR-146b or control miRNA. A statistically significant decrease in *RARB* mRNA expression was obtained for miR-146a-5p (31%, p < 0.0001) and miR-146b (33%, p < 0.0001) (Fig 5). Also, the decrease of RARβ protein has been shown for miR-146a-5p and miR-146b (59% and 15%, respectively), but with no statistical significance (Fig 5C and 5D) that can be due to low sensitivity of the Western Blotting method.

### Inhibition of the miR-146 Family Restores *RARB* Expression in PTC-Derived Cell Line

**Fig 6:**
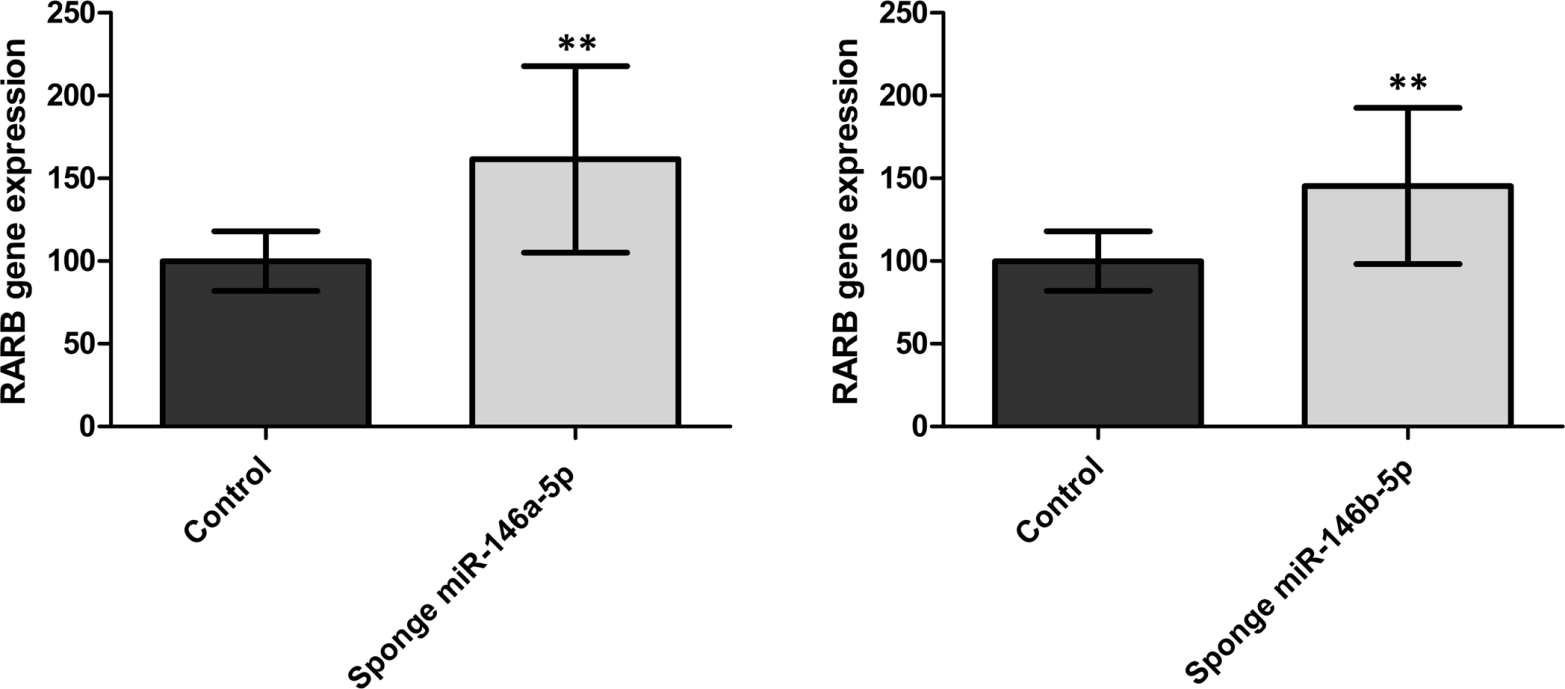
Induction of RARB is shown by a real-time PCR Taqman assay in the K1 cell line after inhibition of endogenous miR-146a-5p (C) or miR-146b-5p (D) by means of transfection with sponge plasmids. Data are expressed as mean values +/- SEM. Statistical analysis was performed using an unpaired t test (** p< 0.01).

The pGL3-Control plasmids containing two tandem sequences 100% complementary to mature miR-146a or miR-146b (pGL3-sponge-miR-146a-5p or pGL3-sponge-miR-146b-5p, respectively) were transfected to the PTC-derived K1 cell lines with low expression of *RARB* and high expression of the miR-146 family. Expression of *RARB* measured by real-time PCR Taqman assay revealed a 62.5% (p = 0.0015) and 45.4% (p = 0.0064) increase of *RARB* expression after inhibition of miR-146a and miR-146b, respectively (Fig 6).

### Inhibition of the miR-146 Family Decreases Proliferation of the PTC-Derived Cell Line

**Fig 7:**
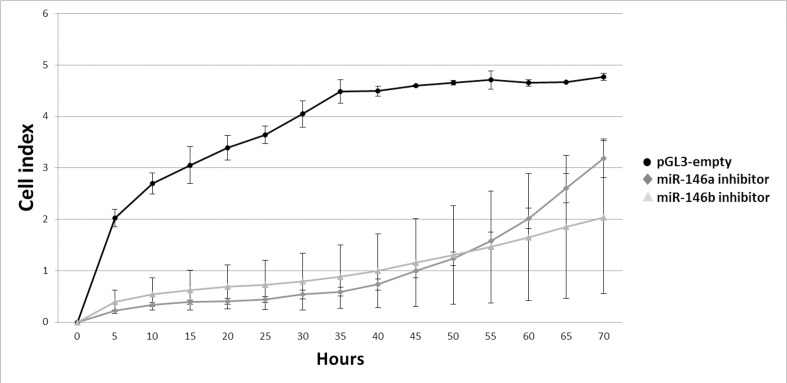
Effect of inhibition of miR-146a-5p and miR-146b-5p on the intensity of cell division. Proliferation of K1 cells transfected with pGL3-sponge-miR-146a-5p (dark gray) or pGL3-sponge-miR-146b-5p (light gray) was analyzed by the xCELLigence system, operating on the basis of electrical resistance measurement. Resistance was measured every 30 minutes for 80 hours. The final cell index values show the difference between the resistance generated by the cells in each time point and the resistance of the medium without cells. The graph indicates proliferation index at each time point and SD for each measurement

An inhibition of endogenous miR-146a-5p or miR-146b-5p by means of transfection of the K1 cell line with sponge plasmids resulted in a decreased proliferation rates of the cells (Fig 7), analyzed using the xCELLigence system. The system measures the electrical resistance of cells that adhere to electrodes on the surface.

## Discussion

Deregulation of microRNAs and a resulting aberrant expression of their target genes is an important factor in the process of carcinogenesis. The aim of this study was to determine the role of miR-146 family in inhibition of the *RARB* gene in papillary thyroid carcinoma. The fact that *RARB* is diminished in a large number of cancers, including thyroid cancer, shows its importance for cellular homeostasis and indicates its tumor suppressive role, which is exerted mainly by the action of its ligand, retinoic acid. The anti-cancer effect of retinoic acid was already shown in numerous studies, including testicular teratoma or head and neck cancer cells, in which retinoic acid treatment stimulates differentiation and apoptosis [20, 21]. Retinoic acid used in patients with thyroid cancer caused inhibition of tumor growth in 65% of patients [22], moreover, retinoic acid was shown to re-induce radioactive iodine uptake in patients refractory to this kind of therapy [10, 22–25]. This function of retinoic acid is possibly mediated by increasing the levels of sodium–iodine symporter [26] that restores the ability of cells to concentrate radioactive iodide [25–28]. An increased expression of *NIS* mRNA and significantly elevated uptake of radioactive iodine after treating cells with retinoic acid were shown in breast cancer [28], anaplastic and medullary thyroid carcinoma [25, 27].

However, low expression of RARβ is a major limitation factor for efficiency of therapies with retinoic acid. So far, mechanisms leading to reduction of *RARB* expression in cancer tissues have not been thoroughly understood. Known modifications of *RARB* include hypermethylation of the gene promoter [29] or loss of 3p chromosome as in the case of non-small cell lung carcinoma [30]. Since thyroid carcinogenesis is accompanied by aberrances in miRNA expression and a consequent deregulation of their target genes, we analyzed the implication of this process in silencing *RARB* in thyroid tumors. *In silico* analysis revealed that the top upregulated miR-146 family potentially bind 3’UTR of *RARB*. Importantly, miR-146 family is significantly involved in pathogenesis of PTC, as the expression of these miRs is severely elevated in PTC [15] and they play important role in PTC predisposition and development [17, 31].

The expression of miRs can be modulated, thus they can serve as promising therapeutic targets in cancer. In this study we showed that inhibition of miR-146a and miR-146b results in restoration of *RARB* and in decrease of proliferation rates of thyroid cancer cells. Previous studies on modification of RARβ expression in hepatoma cells revealed that in the presence of RARβ the cells cease proliferation and begin differentiation [32]. Restoration of *RARB* gene expression in cervical cancer [33], breast cancer [34, 35] and lung cancer [36–38] resulted in inhibition of cell growth and promotion of apoptosis. The same effect was obtained after treatment of cells with retinoic acid [33–38], but only in the presence of sufficient *RARB* levels. Breast cancer cells in which *RARB* was inhibited by siRNA continued to migrate after treatment with retinoic acid, indicating the inability of cells to respond on retinoic acid in the lack of RARβ receptor [39]. These results confirm that the proper level of *RARB* is critical for the efficacy of cancer therapy with retinoic acid. Our study is the first to show the regulation of *RARB* by microRNA, and indicates that understanding of this interaction might provide a new insight into the molecular mechanisms leading to development of papillary thyroid carcinoma. Low levels of RARβ are potentially one of the mechanisms leading to reduced uptake of iodine by tumor cells and ineffectiveness of radioiodine therapy, significantly worsening the prognosis of thyroid cancer patients. By increasing *RARB* levels, microRNA inhibitors may become part of an adjuvant therapy in thyroid cancer patients.

## Supporting Information

S1 Fig A. RARB, miR-146a and miR-146b expression in HeLa, A549 and K1 cell lines. The data are medians with +/- SD (95% CI). RARB mRNA expression was normalized to the reference gene GAPDH, the expression of miR-146a-5p and miR-146b-5p normalized to RNU6B gene. SQ-PCR reaction for each sample was performed in triplicate. B. MiR-146a-5p and miR-146b-5p in non-transfected HeLa cells and after transfection with pcDNA3-miR-146a-5p, or pcDNA3-miR-146b-5p vectors normalized to RNU6B. The results are from three independent experiments. The data are medians with +/- SD (95% CI). Statistical analysis was performed using the Mann Whitney U-test (***P <0.001). C. The level of *RARB* mRNA expression in A549 cells after retinoic acid (ATRA) treatment for 2, 5 and 7 days. The data were normalized against *GAPDH* gene and are shown as mean and SEM. Statistical analysis was performed using an unpaired t test (*** *P* < 0.001).
(PDF)

S1 Table Ct values of the reference genes (*U6B*, *GAPDH*) used in the study (PDF)

S2 Table The sequences of primers used in the study. (PDF)
