# Supplementary material for: Family of microRNA-146 Regulates RARβ in Papillary Thyroid Carcinoma
Source: PLoS One. 2016 Mar 24;11(3):e0151968. doi: 10.1371/journal.pone.0151968 (PMC4807079; doi:10.1371/journal.pone.0151968)
Supplement: S1 Fig — A. RARB, miR-146a and miR-146b expression in HeLa, A549 and K1 cell lines. The data are medians with +/- SD (95% CI). RARB mRNA expression was normalized to the reference gene GAPDH, the expression of miR-146a-5p and miR-146b-5p normalized to RNU6B gene. SQ-PCR reaction for each sample was performed in triplicate. B. MiR-146a-5p and miR-146b-5p in non-transfected HeLa cells and after transfection with pcDNA3-miR-146a-5p, or pcDNA3-miR-146b-5p vectors normalized to RNU6B. The results are from three independent experiments. The data are medians with +/- SD (95% CI). Statistical analysis was performed using the Mann Whitney U-test (***P <0.001). C. The level of RARB mRNA expression in A549 cells after retinoic acid (ATRA) treatment for 2, 5 and 7 days. The data were normalized against GAPDH gene and are shown as mean and SEM. Statistical analysis was performed using an unpaired t test (*** P < 0.001). (PDF) [file pone.0151968.s001.pdf]

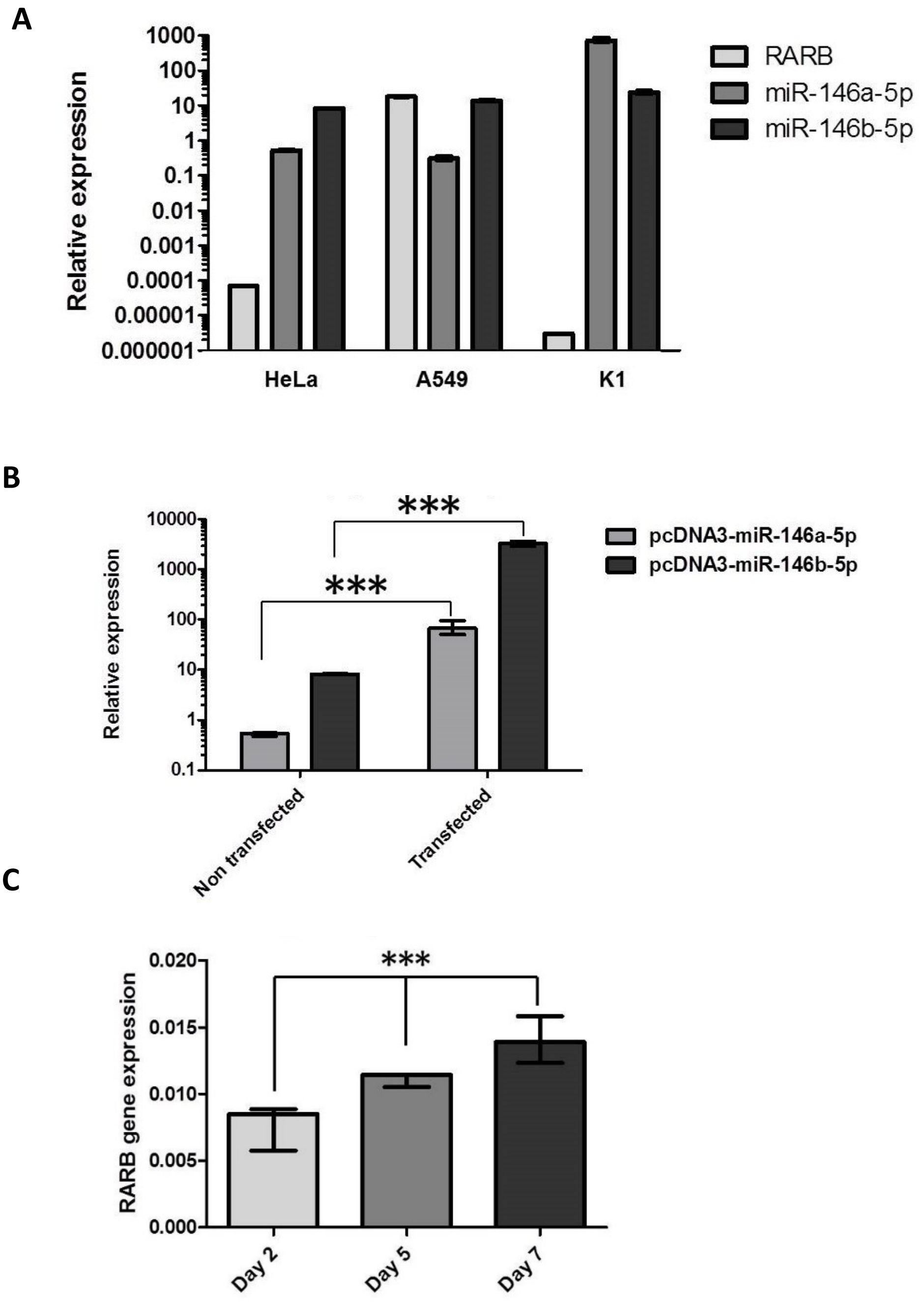

Supplementary Figure 1. A. RARB, miR-146a and miR-146b expression in HeLa, A549 and K1 cell lines. The data are medians with +/- SD (95% CI). RARB mRNA expression was normalized to the reference gene GAPDH, the expression of miR-146a-5p and miR-146b-5p normalized to RNU6B gene. SQ-PCR reaction for each sample was performed in triplicate. B. MiR-146a-5p and miR-146b-5p in non-transfected HeLa cells and after transfection with pcDNA3-miR-146a-5p, or pcDNA3-miR-146b-5p vectors normalized to RNU6B. The results are from three independent experiments. The data are medians with +/- SD (95% CI). Statistical analysis was performed using the Mann Whitney U-test (****P* <0.001). C. The level of *RARB* mRNA expression in A549 cells after retinoic acid (ATRA) treatment for 2, 5 and 7 days. The data were normalized against *GAPDH* gene and are shown as mean and SEM. Statistical analysis was performed using an unpaired t test (*** $P < 0.001$).
