# Supplementary figures and images for: Family of microRNA-146 Regulates RARβ in Papillary Thyroid Carcinoma
Source: PLoS One. 2016 Mar 24;11(3):e0151968. doi: 10.1371/journal.pone.0151968 (PMC4807079; doi:10.1371/journal.pone.0151968)

| Patient ID | U6B-PTC-N | U6B-PTC-T | GAPDH-PTC-N | GAPDH-PTC-T |
|------------|-----------|-----------|-------------|-------------|
| 537        | 29.21     | 27.29     | 24.77       | 25.03       |
| 548        | 30.83     | 29.63     | 25.98       | 26.67       |
| 1501       | 26.43     | 26.43     | 26.79       | 25.73       |
| 1510       | 28.82     | 28.61     | 26.47       | 29.77       |
| 1517       | 28.02     | 28.22     | 26.92       | 25.56       |
| 1531       | 26.96     | 27.46     | 26.17       | 25.36       |
| 1532       | 31.01     | 30.64     | 26.44       | 29.28       |
| 1538       | 25.32     | 27.22     | 30.1        | 24.77       |
| 1541       | 26.86     | 25.73     | 30.42       | 28.37       |
| 1543       | 28.65     | 29.03     | 28.26       | 26.49       |
| 1544       | 27.33     | 27.77     | 30.85       | 24.68       |
| 1546       | 26.87     | 26.82     | 27.52       | 26.94       |
| 1547       | 27.5      | 27.07     | 26.06       | 25.76       |
| 1556       | 24.68     | 29.91     | 26.16       | 26.99       |
| 1557       | 29.14     | 28.57     | 27.87       | 25.31       |
| 1560       | 28.09     | 28.1      | 27.13       | 26.36       |
| 1571       | 25.02     | 27.1      | 25.97       | 26.95       |
| 1580       | 29.36     | 29.08     | 28.01       | 27.91       |
| 1584       | 29.24     | 29.4      | 26.02       | 25.58       |
| 1604       | 26.52     | 27.42     | 24.61       | 23.9        |
| 1614       | 27.55     | 28.88     | 30.64       | 26.5        |
| 1622       | 30.1      | 28.38     | 33.77       | 26.01       |
| 1624       | 28.05     | 28.56     | 26.62       | 25.96       |
| 1629       | 30.23     | 29.38     | 26.27       | 25.82       |
| 1632       | 26.45     | 27.51     | 26.06       | 23.77       |
| 1644       | 32.63     | 33.22     | 27.43       | 25.68       |
| 1645       | 27.47     | 30.25     | 28.03       | 25.47       |
| 1653       | 31.96     | 30.53     | 29.36       | 26.14       |
| 1673       | 32.36     | 31.7      | 26.96       | 27.53       |
| 1683       | 28.77     | 28.85     | 27.27       | 27.9        |
| 1691       | 26.6      | 28.2      | 29.11       | 28.1        |
| 1693       | 25.87     | 28.74     | 28.6        | 26.24       |
| 1694       | 29.76     | 26.21     | 27.41       | 28.55       |
| 1697       | 30.08     | 27.55     | 28.91       | 27.9        |
| 1700       | 30.25     | 30.2      | 26.02       | 27.23       |
| 1705       | 30.42     | 27.07     | 27.01       | 27.14       |
| 1711       | 27.76     | 27.87     | 26.86       | 27.08       |
| 1715       | 29.03     | 26.64     | 28.57       | 28.29       |
| 1719       | 28.83     | 30.62     | 26.82       | 24.83       |
| 1928       | 29.85     | 29.29     | 27.03       | 25.95       |
| 1982       | 32.11     | 29.89     | 26.39       | 25.54       |
| 100029     | 30.22     | 28.86     | 25.65       | 25.27       |
| 10468      | 30.13     | 30.59     | 26.26       | 25.73       |
| 10481      | 30.32     | 28.95     | 26.17       | 25.78       |
| 100043     | 29.33     | 30.99     | 34.2        | 39.73       |
| 1595       | 26.67     | 28.08     | 26.91       | 26.12       |
| 1714       | 24.8      | 29.78     | 26.72       | 24.22       |
| 1521       | 28.3      | 30.94     | 26.19       | 26.4        |

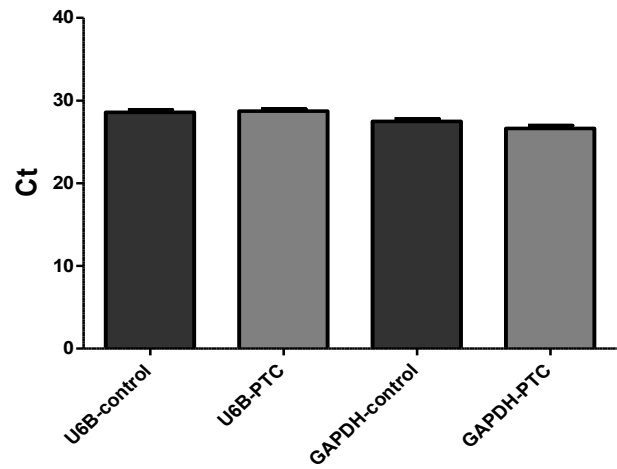

Supplement: S1 Table — (PDF) [file pone.0151968.s002.pdf]
