# Supplementary material for: Family of microRNA-146 Regulates RARβ in Papillary Thyroid Carcinoma
Source: PLoS One. 2016 Mar 24;11(3):e0151968. doi: 10.1371/journal.pone.0151968 (PMC4807079; doi:10.1371/journal.pone.0151968)
Supplement: S2 Table — (PDF) [file pone.0151968.s003.pdf]

Supplementary Table 2. The sequences of primers used in the study.

**pGL3-RARB Construct**	
RARB-Forward	ACGCTCTAGAGTCACCACTCGTGCAATAAGAC
RARB-Reverse	AACGTCTAGACTTTGCCAGGAGACTCCATG
**pGL3-sponge-miR Constructs**	
pGL3-sponge_miR Forward_PstI	AATTCTGCAGGTCACCACTCGTGCAATAAGAC
pGL3-sponge_miR Reverse_KpnI	AATTGGTACCCTTTGCCAGGAGACTCCATG
**Mutagenesis**	
miR-146a-5p	
Forward primer	GGTTGTGTCAGTGTCAGACCCTCGAAATTCAGTTCTTCAGCTG
Reverse primer	CAGCTGAAGAACTGAATTTCGAGGGTCTGACACTGACACAACC
miR-146a-3p	
Forward primer	CGATGTGTATCCTCAGCTTGACCCACTGAATTCCATGGGTTGTG
Reverse primer	CACAACCCATGGAATTCAGTGGGTCAAGCTGAGGATACACATCG
**Sponges sequences**	
Sponge_miR-146a	CACTCGTGCAATAAGACAGCAAGGCTCAGTCCATCTCTAGAAGCAAGGCTCAGTCCATCTCTAGACATGGAGTCTCCTGG
sponge_miR-146b	CACTCGTGCAATAAGACACAAAAGCGGTGCTATCCTTAGGTACAAAAGCGGTGCTATCCTTAGGTCATGGAGTCTCCTGG
